# Supplementary material for: Sensory Modulation in Children with Developmental Coordination Disorder Compared to Autism Spectrum Disorder and Typically Developing Children
Source: Brain Sci. 2022 Aug 31;12(9):1171. doi: 10.3390/brainsci12091171 (PMC9496992; doi:10.3390/brainsci12091171)
Supplement: Supplementary file 1 [file brainsci-12-01171-s001.zip › brainsci-1878457-supplementary.pdf]

Supplementary Table S1. Cohen's d effect size for paired comparisons between subject groups.

	DCD:TD	DCD:ASD	TD:ASD
	Cohen's d	*Cohen's d*	*Cohen's d*
Age	0	0.06	-0.06
Full-Scale IQ	0.58	-0.13	0.70
DCD/ASD>TD or TD>DCD/ASD			
SSP-2 Bystander	-1.32	0.38	-1.60
DCDQ Total	3.08	0.20	2.97
DCD between ASD and TD: ASD>DCD>TD or TD>DCD>ASD			
SSP-2 Seeker	-0.98	0.67	-1.52
SSP-2 Avoider	-1.02	1.08	-2.10
SSP-2 Sensor	-1.23	1.27	-2.37
CBCL Social Problems	-1.57	-1.03	-1.76
CBCL Attention Problems	-1.84	0.68	-1.61
CBCL Thought Problems	-0.97	0.98	-1.80
CBCL Total Competencies	0.70	-0.98	1.78
CBCL School Competencies	1.39	-0.61	2.02
ASD>DCD/TD or DCD/TD>ASD			
SensOR Total	-0.83	1.22	-2.03
SensOR Tactile	0.69	1.17	-1.80
SensOR Auditory	-0.63	1.04	-1.70
RBS Stereotyped	-1.20	0.94	-1.64
RBS Compulsive	0.07	0.94	-1.00
RBS Ritualistic	-0.23	1.44	-1.69
RBS Sameness	-0.61	1.24	-1.66
RBS Restricted	-0.68	1.12	-1.62
RBS Total	-0.67	1.30	-1.72
CBCL Anxious/Depressed	-0.25	0.86	-1.15
CBCL Withdrawn/Depressed	-0.74	0.84	-1.46
CBCL Somatic Complaints	-0.42	0.72	-1.10
CBCL Social Competencies	0.34	-1.03	1.43
CBCL Aggressive Behavior	-0.82	0.56	-1.01
SCARED- P GAD	-0.40	0.68	-1.16
SCARED-P Social Anxiety	0.08	0.91	-0.84
SCARED-P Separation Anxiety	-0.12	0.85	-1.02
SCARED-P School Avoidance	-0.32	0.44	-0.71
SCARED- P Panic	-0.21	0.71	-0.94
SCARED- P Total [b]	-0.24	0.92	-1.23
NEPSY-ToM	0.25	0.79	1.09
Alexi-Describing Feelings	0.26	0.95	-0.62

	DCD:TD	DCD:ASD	TD:ASD
	Cohen's d	*Cohen's d*	*Cohen's d*
Alexi- 2 factor total	0.05	0.63	-0.56
ASD>TD only			
RBS Self Injurious	-0.37	0.46	-0.74
CBCL Rule Breaking Behavior	-0.75	0.28	-0.84
IRI Personal Distress	0.20	0.24	-0.45
TD>ASD only			
CBCL Activities Competencies	0.33	-0.49	0.87
No significant differences between groups			
IRI Perspective Taking	0.02	-0.37	0.39
IRI Fantasy Scale	-0.11	-0.14	0.30
IRI Empathetic Concern	-0.11	-0.32	0.22
NEPSY- Affect Recognition	0.12	-0.49	0.62
Alexi- Identifying Feelings	-0.13	0.28	-0.41

IQ= Intelligence Quotient; SSP= Short sensory Profile; DCDQ= Developmental Coordination Disorder Questionnaire; CBCL= Child Behavior Checklist; SenSOR= Sensory Over-Responsivity Inventory; RBS= Repetitive Behaviors Scale; SCARED-P= SCARED Parent; GAD= Generalized Anxiety Disorder; NEPSY= Neuropsychological Assessment; ToM= Theory of Mind; Alexi= Alexithymia; IRI= Interpersonal Reactivity Index
